# Characterization of the Rumen Microbiota and Volatile Fatty Acid Profiles of Weaned Goat Kids under Shrub-Grassland Grazing and Indoor Feeding

**DOI:** 10.3390/ani10020176

**Published:** 2020-01-21

**Authors:** Jiazhong Guo, Pengfei Li, Shuai Liu, Bin Miao, Bo Zeng, Yahui Jiang, Li Li, Linjie Wang, Yu Chen, Hongping Zhang

**Affiliations:** 1 College of Animal Science and Technology, Sichuan Agricultural University, Chengdu 611130, China; jiazhong.guo@sicau.edu.cn (J.G.); lipengfei@stu.sicau.edu.cn (P.L.); liushuaigg888888@sina.com (S.L.); apollobovey@163.com (B.Z.); jyh@sicau.edu.cn (Y.J.); lily@sicau.edu.cn (L.L.); wanglinjie@sicau.edu.cn (L.W.); 2 Nanjiang Yellow Goat Scientific Research Institute, Nanjiang 635600, China; miaobin1435@163.com (B.M.); 8222074@163.com (Y.C.); 3 Farm Animal Genetic Resources Exploration and Innovation Key Laboratory of Sichuan Province, Sichuan Agricultural University, Chengdu 611130, China

**Keywords:** goat, rumen, microbiota, volatile fatty acid, grazing, 16S rRNA gene

## Abstract

**Simple Summary:**

Although grazing and indoor feeding are both major production systems in the goat industry worldwide, the impacts of different feeding systems on rumen fermentation remain poorly understood. In this study, we observed large differences in microbial community compositions and volatile fatty acid profiles in the rumen of weaned goats among three feeding systems, which provides an in-depth understanding of rumen fermentation in response to changes in feeding systems.

**Abstract:**

In this study, we conducted comparative analyses to characterize the rumen microbiota and volatile fatty acid (VFA) profiles of weaned Nanjiang Yellow goat kids under shrub-grassland grazing (GR), shrub-grassland grazing and supplementary feeding (SF), and indoor feeding (IF) systems. We observed significant differences (*p* < 0.05) in the concentrations of total VFA and the proportions of acetate and butyrate in the rumen fluid among the three groups, whereas the proportions of propionate and the acetate/propionate ratio did not differ substantially. Alpha diversity of the rumen bacterial and archaeal populations in the GR and SF kids was significantly higher (*p* < 0.05) than that in the IF goat kids, and significant differences (*p* < 0.05) in similarity were observed in the comparisons of GR vs. IF and SF vs. IF. The most predominant bacterial phyla were Bacteroidetes and Firmicutes across the three groups, and the archaeal community was mainly composed of Euryarchaeota. At the genus and species levels, the cellulose-degrading bacteria, including *Lachnospiraceae*, *Ruminococcaceae* and *Butyrivibrio fibrisolvens*, were abundant in the GR and SF groups. Furthermore, 27 bacterial and 11 unique archaeal taxa, such as *Lachnospiraceae*, *Butyrivibrio fibrisolvens*, and *Methanobrevibacter ruminantium*, were identified as biomarkers, and showed significantly different (*p* < 0.05) abundances among the three groups. Significant Spearman correlations (*p* < 0.05), between the abundances of several microbial biomarkers and the concentrations of VFAs, were further observed. In summary, our results demonstrated that the adaptation to grazing required more rumen bacterial populations due to complex forage types in shrub-grassland, although the rumen fermentation pattern did not change substantially among the three feeding systems. Some microbial taxa could be used as biomarkers for different feeding systems, particularly cellulose-degrading bacteria associated with grazing.

## 1. Introduction

In the gut of ruminants (e.g., cattle, sheep and goats), the rumen is a major habitat for microorganisms, consisting of a wide variety of anaerobic bacteria, archaea, fungi and protozoa [1,2]. Strikingly, many ruminal microbes are capable of efficiently degrading fibrous feedstuffs through the actions of the enzymes they produce and converting feed to volatile fatty acids (VFAs) [3,4], which provide an important energy source for their hosts. When ruminants are fed fiber-deficient diets over a long period, the microbial ecology is altered, and the animal becomes more susceptible to metabolic disorders [1]. Furthermore, archaeal populations (i.e., methanogens) of the rumen utilize CO_2_ and H_2_ as the main substrates to produce methane, thereby eliminating the inhibitory effect of hydrogen upon fermentation, but resulting in a loss of dietary energy [5].

Although there is a core microbial community in the rumen [6,7], a large number of studies have reported that the rumen microbiome can be changed drastically by many factors, such as age [8,9,10], dietary source [11,12], feeding system [13,14,15], host species [7] and even geography [7]. For example, Wang et al. investigated the temporal dynamics of the rumen microbiome in goats, and observed that the relative abundance of Firmicutes did not vary with age [9]. Considering the dietary composition or source, the rumen bacterial diversity increased in goats that were fed a forage diet compared to a mixed forage-concentrate diet [16]. Similarly, the relative abundance of Bacteroidetes decreased significantly in the rumen of cashmere goats when the dietary forage to concentrate ratio was decreased [17]. A recent study also revealed that a high-grain diet resulted in an increase in ruminal acidity and a very high Firmicutes/Bacteroidetes ratio (~3:1) in goats [18], which are thought to be unhealthy changes. It was also demonstrated that administering antibiotics decreased the rumen bacterial diversity in goats [19]. Taken together, these studies characterized the rumen microbial population dynamics of goats under indoor feeding conditions, whereas few studies have reported the rumen microbiome in goats under grazing conditions [19].

To reduce feed costs and protect animal welfare, grazing is a very important production system for the goat industry worldwide, particularly in mountainous regions. To date, the rumen microbiome has been compared in cattle [13], sheep [14] and yaks [15] under grazing and indoor conditions, mainly reflecting a mixed effect of dietary source and forage to concentrate ratios. Although the rumen microbiota of goats fed in pastures was characterized based on a limited amount of 16s rRNA sequence data [20], no study has compared the rumen microbial diversity and composition in goats under different feeding systems until now.

The Nanjiang Yellow goat is a breed developed for meat production that is widely distributed in Southwest China. Indoor feeding is the major goat production system in the plains of Southwest China, whereas grazing is the dominant feeding regime in the mountains (e.g., the Qinba Mountains) of this region. However, the rumen microbiome in Nanjiang Yellow goats has not yet been reported, regardless of indoor feeding or grazing. In this study, we performed comparative analyses to characterize the rumen microbiota and VFA profiles of weaned Nanjiang Yellow goat kids under different feeding systems.

## 2. Materials and Methods

### 2.1. Ethics Statement

The experiments involving animals in this study were conducted in agreement with the guidelines and regulations for the Administration of Affairs Concerning Experimental Animals (Ministry of Science and Technology, China). All experimental protocols were approved by the Institutional Animal Care and Use Committee of the College of Animal Science and Technology, Sichuan Agricultural University (No. DKYB20081003).

### 2.2. Experimental Design and Collection of Rumen Fluid

In the present study, the feeding trial was conducted during September and November 2018 on the Nanjiang Yellow goat breeding farm in Beiji of Nanjiang county (i.e., in the Qinba Mountains), Sichuan, China (~1000 m altitude; 107.00° E, 32.16° N), since goat kids are mainly born from May to June in each year and are weaned at ~60 days of age on the farm. A total of 90 weaned Nanjiang Yellow goat kids, all about three months old, were selected for the feeding experiment (average body weight 13.25 ± 1.05 kg, male sex), and were randomly assigned into three feeding systems, namely, the grazing group (GR, n = 30), the grazing and supplementary feeding group (SF, n = 30), and the indoor feeding group (IF, n = 30). The grassland in the Qianba Mountains is a subtropical shrub-grassland and the vegetation in this region mainly includes various perennial grasses, such as *Imperata cylindrica*, *Miscanthus sinensis* and *Deyeuxia arundinacea*, and fodder shrubs, such as *Lespedeza bicolor* and *Indigofera amblyantha*. According to nutritional requirements (NY/T816-2004, Ministry of Agriculture and Rural Affairs, China), a concentrate supplement was formulated for the goat kids in the IF and SF groups (Table S1), and the forage was composed of sorghum–sudangrass hybrid silages. The kids in the IF group were fed a diet with a forage to concentrate ratio of 66:34 three times per day at 07:00, 14:00 and 18:00, whereas the kids in the SF and GR groups were grazed on shrub-grasslands that also consisted of shrubs and grasses for 7 and 11 h, respectively. After grazing, the kids in the SF group were supplemented with the same diet as the kids in the IF group.

On day 60, more than 50 mL of original rumen digesta was randomly collected from six kids in each group, using a stomach tube attached to a vacuum pump before the morning feeding. Approximately 30 mL of rumen fluid was subsequently obtained by squeezing the digesta through four layers of cheesecloth. All samples were snap-frozen in liquid nitrogen and stored at −80 °C until DNA extraction.

### 2.3. Measurement of VFAs and DNA Extraction

Each rumen fluid sample of 5 mL was diluted with 1 mL deproteinizing solution (25% orthophosphoric acid) to determine the concentration of VFAs. Each sample was pre-processed with 25% (*w/w*) metaphosphoric acid. Then, the supernatant was measured with the Agilent-6890 (Santa Clara, CA, USA) NGC system using a Thermon-3000 5% Shincarbon A column at 190 °C.

The microbial cells were separated from 1.5 mL of rumen fluid and metagenomic DNA was extracted with the OMEGA Stool DNA Kit following the manufacturer’s protocol (Omega Bio-Tek Inc., Norcross, GA, USA). DNA quality was assessed via 1% agarose gel electrophoresis, and DNA concentrations were determined using a NanoDrop 2000 spectrophotometer (Thermo Scientific, Waltham, MA, USA).

### 2.4. 16S rRNA Gene Sequencing

To accurately analyze the bacterial community, the full-length fragment of the 16S rRNA gene was amplified by polymerase chain reaction (PCR) for Single-molecule real-time sequencing (SMRT) using a primer pair (F: 5′-AGAGTTTGATCCTGGCTCAG-3′; R: 5′-GNTACCTTGTTACGACTT-3′). The PCR program was as follows: 95 °C for 2 min; 35 cycles of 95 °C for 30 s, 60 °C for 45 s and 72 °C for 90 s, with a final extension of 72 °C for 10 min (Hou et al., 2015). The barcoded PCR products were resolved by 2% agarose gel electrophoresis, and were purified with a Qiaquick PCR purification kit (Qiagen, Valencia, CA, USA). The SMRT Bell libraries were finally sequenced using the Pacific Biosciences (PacBio) Sequel system (Novogene Co., Ltd., Beijing, China).

To better understand the archaeal community, the V7–V8 region of the 16S rRNA gene was amplified using a primer pair (1106F: 5′-TTWAGTCAGGCAACGAGC-3′ and 1378R: 5′-TGTGCAAGGAGCAGGGAC-3′). After the barcoded PCR products were purified, the library for each sample was constructed and was subjected to single-end sequencing on the IonS5TMXL platform (Novogene).

All raw amplicon sequence data in this study are available at the National Center for Biotechnology Information (NCBI) SRA Science Research Associates (SRA) database under accession: PRJNA593344.

### 2.5. Data Analysis

Raw sequencing reads were processed to remove adapter sequences using cutadapt [21] (v1.9.1), and the chimeric sequences were filtered with UCHIME [22] for PacBio long reads and VSEARCH [23] for short reads, respectively. We only retained the PacBio long reads with the expected amplicon length of 1240–1540 nt for downstream analyses. The high-quality reads were clustered into operational taxonomic units (OTUs) at a 97% sequence similarity threshold, using Uparse [24] (v7.0), and taxonomic assignments of the OTUs (number of reads ≥ 2) were performed using the classify.otu command in Mothur [25] by comparison with the SILVA database (v132). After a de novo taxonomic tree was constructed using MUSCLE [26] (v3.8.31), alpha and beta diversity measurements were performed using QIIME [27] (v1.9.1). The reads assigned to any known bacterial taxa were deleted during the archaeal community analyses.

To identify the specific microbial taxa associated with the three feeding regimes, we conducted a pairwise comparison of the rumen microbiota in the three groups using the linear discriminant analysis (LDA) effect size (LEfSe) with default parameters [28] (LDA score > 4), which would allow the discovery of biomarkers. To integrate the rumen microbiota and VFAs, Spearman correlation analysis was carried out between the identified biomarkers and the VFAs in each of the feeding systems using R software [29]. Only correlations with *p* < 0.05 were considered to be significant.

## 3. Results

### 3.1. The Concentrations of VFAs Among the Three Feeding Systems

As shown in Table 1, the concentration of total VFA in the IF group was significantly much higher (*p* < 0.01) than that in the other two feeding systems. The highest proportion of acetate (*p* = 0.011) was also observed in the IF group, whereas the molar proportions of propionate and the acetate/propionate ratio in the rumen fluid did not differ among the three feeding systems. Furthermore, the SF-fed goat kids had the largest proportion of ruminal butyrate (*p* < 0.01), while the largest fractions of the remaining types of VFAs were found in the rumen fluid of the GR-fed goat kids.

### 3.2. Rumen Microbial Diversity and Similarities Among the Three Feeding Systems

After quality control of 304,483 PacBio raw sequences, 239,170 high-quality long reads (total average length of 1433 nt) were obtained for further analyses, with an average of 13,287 reads per sample (Table S2). To better understand the rumen archaeal community, 1,172,712 high-quality short reads (total average length of 278 nt) were obtained from all samples with an average of 65,150 reads per sample (Table S2). Accordingly, a total of 1588 and 465 OTUs were identified for bacteria and archaea across all samples, respectively.

GR and SF had significantly higher bacterial community richness than IF (*p* = 0.02), as measured by the number of observed species (Table 2). Similarly, the Shannon diversity index in SF (5.88) was significantly higher (*p* = 0.04) than that in IF (4.56). No significant differences in alpha diversity were observed between GR and SF, based on four indices (Table 2). The Chao1 indices indicated that the alpha diversities of the archaeal populations in the GR and SF groups were significantly higher than that in the IF group (*p* = 0.01), whereas no significant differences (*p* > 0.05) in diversity were observed among the three feeding systems using the Shannon and Simpson indices.

**Figure 1:**
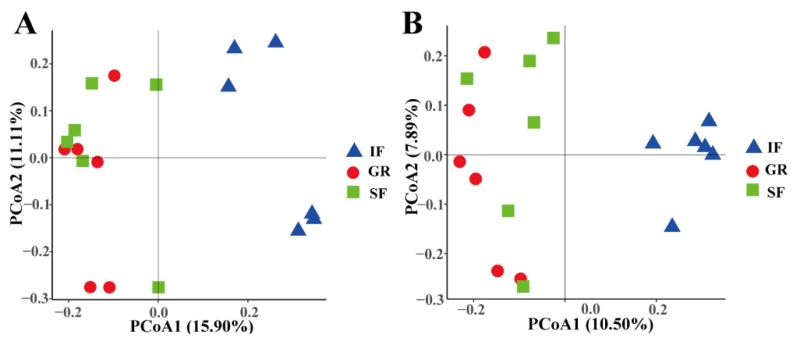
Principal coordinate analysis (PCoA) plots of the bacterial and archaeal community compositions in the rumen fluid of the goat kids among the three feeding systems using an unweighted UniFrac metric. The percentages of variation explained by PC1 and PC2 are indicated on the axes. (**A**) The PCoA plot of the bacterial community composition; (**B**) the PCoA plot of the archaeal community composition.

According to the bacterial and archaeal community comparisons using an unweighted UniFrac metric, the rumen microbial samples in the GR and SF groups gathered closely into a large group along the first principal coordinate (variance explained = 15.90% for bacteria and 10.50% for archaea), whereas the IF group samples were tightly clustered (Figure 1). The analysis of similarity (ANOSIM) further indicated that there were significant differences in the bacterial community compositions of GR vs. IF (R = 0.96, *p* = 0.006) and SF vs. IF (R = 0.89, *p* = 0.002) (Table S3), but not for the comparison of GR vs. SF (R = 0.006, *p* = 0.49). Similar results were also revealed in the comparisons of archaeal populations among the three groups (Table S3).

### 3.3. Rumen Microbial Community Composition Across the Three Feeding Systems

**Figure 2:**
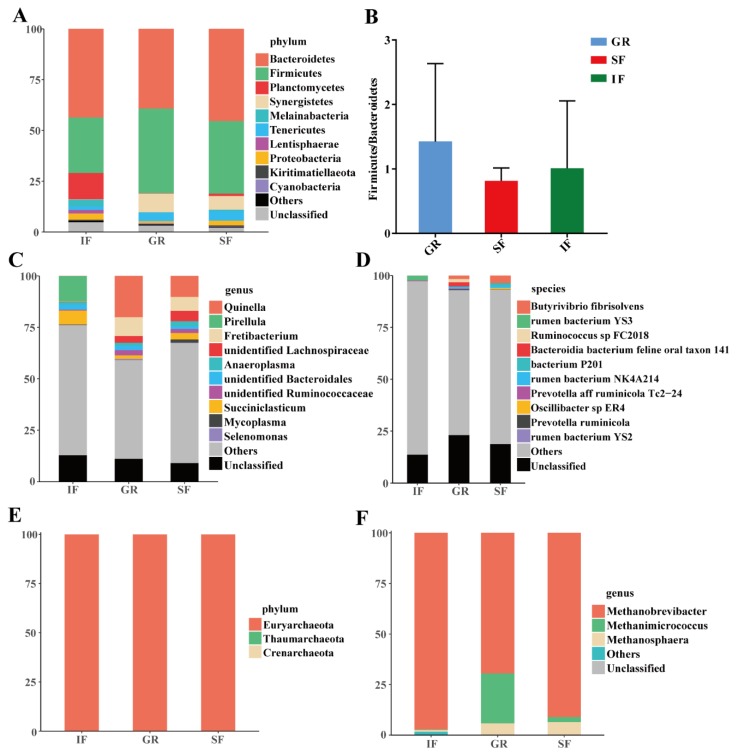
Bacterial and archaeal community compositions at different taxon levels in the rumen fluid of the goat kids across the three feeding systems. (**A**) The composition of bacteria at the phylum level; (**B**) the Firmicutes/Bacteroidetes ratios among the three feeding systems; (**C**) the composition of bacteria at the genus level; (**D**) the composition of bacteria at the species level; (**E**) the composition of archaea at the phylum level; (**F**) the composition of archaea at the genus level. The “Others” proportion represents the known and unidentified taxa with low abundances at different taxon levels.

A total of 18 bacterial phyla were found across all samples (Table S4), and the two most predominant phyla were Bacteroidetes and Firmicutes with proportions of 42.73% and 34.90% on average, respectively (Figure 2A). The phylum Tenericutes also showed high percentages (1.79–4.69%) in the three groups. The remaining abundant phyla in the GR and SF groups mainly included Synergistetes (9.23% and 6.77%), whereas the relative abundances of Planctomycetes, Melainabacteria and Proteobacteria were as high as 12.95%, 3.35% and 3.05% in the IF group, respectively (Figure 2A). As shown in Figure 2B, no significant differences were observed in the ruminal Firmicutes/Bacteroidetes ratios among the three feeding systems.

The three most predominant genera in the GR and SF groups were *Quinella* (20.01% and 10.19%), *Fretibacterium* (9.23% and 6.77%), and *unidentified Lachnospiraceae* (3.27% and 5.06%) (Figure 2C), whereas *Pirellula*, *Succiniclasticum*, and *unidentified Bacteroidales* showed high relative abundances in the IF group, representing 12.35%, 6.85% and 2.46% of the total reads (Figure 2C), respectively.

A total average of 81.52% of the high-quality long reads were classified into 49 known and 1103 unidentified species in the SILVA database (Table S4), suggesting that many ruminal bacteria have not yet been characterized. The proportions of *Bacteroidia bacterium feline oral taxon 141* (2.08%), *Butyrivibrio fibrisolvens* (1.64%), and *Ruminococcus sp FC2018* (1.56%) were relatively high in the GR group. *Butyrivibrio fibrisolvens* (3.57%) and *bacterium P201* (1.19%) were the most abundant species in the SF group. The most abundant sequences were mapped into the *rumen bacterium YS3* (2.31%) in the IF group.

Among the three archaeal phyla identified across the three feeding systems (Table S4), Euryarchaeota was the most predominant phyla and showed an average relative abundance of 99.99% across all samples (Figure 2E). At the genus level, a total of 15 archaeal genera were detected, and *Methanobrevibacter* was the most abundant genus (85.90% on average), followed by *Methanimicrococcus* (9.16%) and *Methanosphaera* (4.26%) (Figure 2F).

### 3.4. Effects of the Feeding Systems on Rumen Microbial Compositions Among the Three Feeding Systems

**Figure 3:**
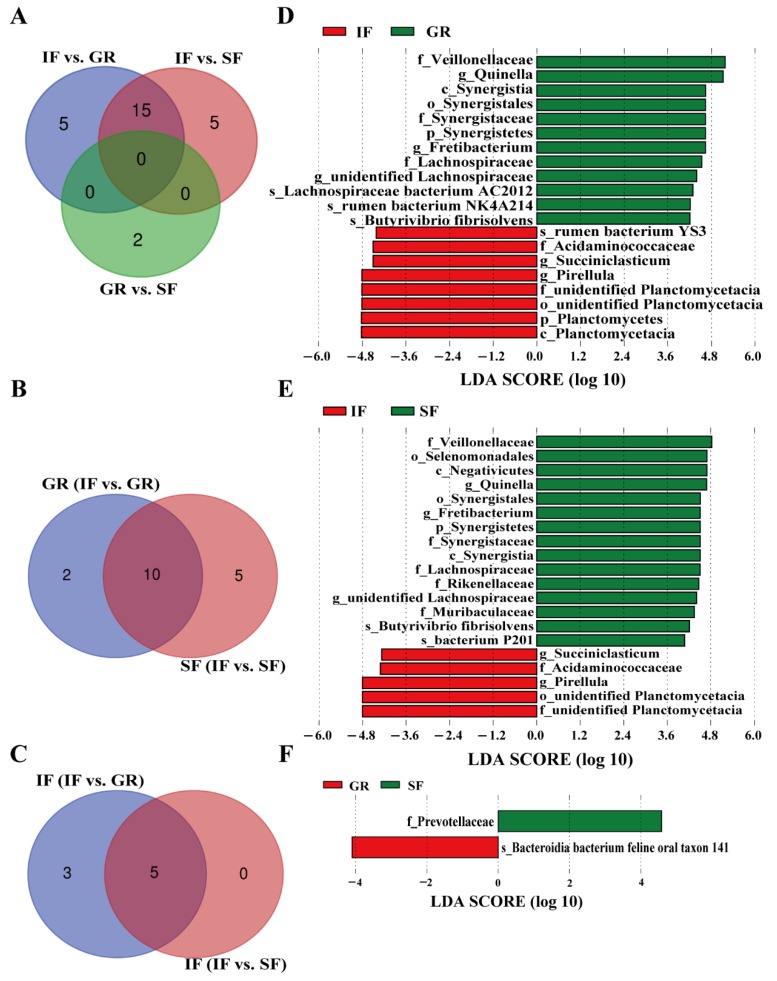
Bacterial taxa with significantly different abundances in each of the three feeding systems based on pairwise comparisons using linear discriminant analysis effect size (LEfSe) (linear discriminant analysis (LDA) score > 4 and *p* < 0.05). (**A**) Number of common and unique biomarkers between pairwise comparisons of the three feeding systems; (**B**) number of common and unique biomarkers in the supplementary feeding (SF) and shrub-grassland grazing (GR) groups based on the pairwise comparisons of GR vs. indoor feeding (IF) and SF vs. IF; (**C**) number of common and unique biomarkers in the IF group based on the pairwise comparisons of GR vs. IF and SF vs. IF; (**D**) the bacterial biomarkers identified in the pairwise comparison between GR and IF; (**E**) the bacterial biomarkers identified in the pairwise comparison between SF and IF; (**F**) the bacterial biomarkers identified in the pairwise comparison between GR and SF.

Based on pairwise comparisons using LEfSe, a total of 27 unique bacterial biomarkers at different taxon levels showed significantly different (LDA score > 4 and *p* < 0.05) abundances in the rumen fluid among the three feeding systems (Figure 3A,D–F). In the pairwise comparisons of GR vs. IF and SF vs. IF, 10 bacterial taxa were detected as common biomarkers in the GR and SF groups (Figure 3B,D,E), particularly cellulose-degrading bacteria, including *Veillonellaceae*, *Lachnospiraceae*, *Butyrivibrio fibrisolven*, and *rumen bacterium NK4A214*. From a phylogenetic point of view, these biomarkers mainly included the phylum Synergistetes and its members (e.g., *Synergistaceae* and *Fretibacterium*) (Figure S1).

In contrast, the phylum Planctomycetes and its members (e.g., *Planctomycetacia* and *Pirellula*) and two members (i.e., *Acidaminococcaceae* and *Succiniclasticum*) of the order *Selenomonadales* made up the majority of biomarkers in the IF group (Figure S1). In the comparison between GR and SF, only *Bacteroidia bacterium feline oral taxon 141* and *Prevotellaceae* were biomarkers in these two groups, respectively, which agreed with the results of the PCoA analysis (Figure 3F).

**Figure 4:**
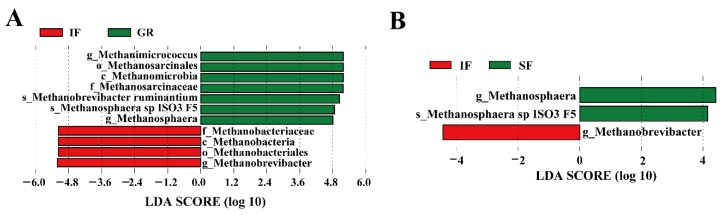
Archaeal taxa showing significantly different abundances in each of the three feeding systems based on pairwise comparisons using LEfSe (LDA score > 4 and *p* < 0.05). (**A**) Archaeal biomarkers identified in the pairwise comparison between GR and IF; (**B**) archaeal biomarkers identified in the pairwise comparison between SF and IF.

A total of 11 unique taxa were identified as biomarkers in the rumen archaeal community (*p* < 0.05) (Figure 4A,B). In the comparison between GR and IF, the class *Methanomicrobia* and its three members and three taxa of hydrogenotrophic methanogens (*Methanosphaera*, *Methanobrevibacter ruminantium*, and *Methanosphaera sp ISO3 F5*) were more abundant biomarkers in GR, whereas only the class *Methanobacteria* and its hydrogenotrophic members (e.g., *Methanobacteriaceae* and *Methanobrevibacter*) were overrepresented as biomarkers in IF (Figure 4A and Figure S1). Two taxa of the methylotrophic methanogens (*Methanosphaera* and *Methanosphaera sp ISO3 F5*) were overrepresented as biomarkers in SF compared to IF, whereas only the genus *Methanobrevibacter* was detected as a biomarker in IF (Figure 4B). Furthermore, none of the archaea taxa showed significantly different abundances between the GR and SF groups.

### 3.5. Spearman Correlations Between Microbial Biomarkers and VFAs in the Rumen Fluid

**Figure 5:**
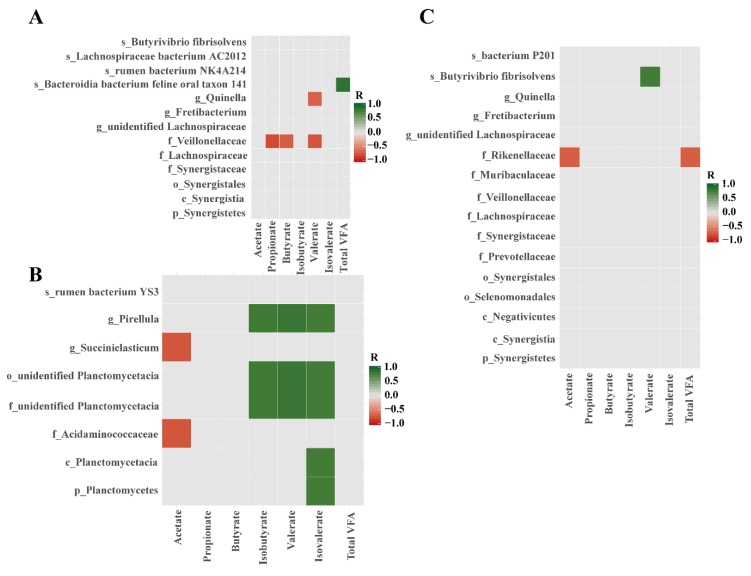
Spearman correlations between bacterial biomarkers and VFAs in the GR (**A**), IF (**B**), and SF (**C**) groups. Correlations with a threshold of statistical significance at *p* < 0.05 were visualized. The green color represents a positive correlation and the red color represents a negative correlation.

Correlation analysis was performed between the 27 bacterial and 11 archaeal biomarkers and VFAs. Most of the microbial biomarkers did not show significant Spearman correlation with VFAs in each feeding system (*p* > 0.05) (Figure 5). The family *Veillonellaceae* was negatively correlated with propionate, butyrate and valerate concentrations, the genus *Quinella* was negatively correlated with the valerate concentration, and the species *Bacteroidia bacterium feline oral taxon 141* was positively correlated with total VFA in the GR group (*p* < 0.05) (Figure 5A). A positive correlation was found between the genus *Pirellula* and isobutyrate, isovalerate, and valerate, whereas the genus *Succiniclasticum* was negatively associated with acetate in the IF group (*p* < 0.05) (Figure 5B). There was a significant positive correlation between *Butyrivibrio fibrisolvens* and valerate (*p* < 0.05), and the family *Rikenellaceae* was negatively correlated with acetate and total VFA (*p* < 0.05) in the SF group (Figure 5C). Furthermore, the species *Methanosphaera sp ISO3 F5* was negatively correlated with propionate, butyrate, valerate and total VFA in the GR group (*p* < 0.05) (Figure S2).

## 4. Discussion

Grazing and indoor feeding are both major production systems in the goat industry worldwide. However, studies regarding the effects of different feeding systems on the rumen fermentation and microbiota are insufficient [20,30]. In this study, we sought to characterize and compare the rumen microbiota and VFA profiles of weaned goat kids under three different feeding systems using 16S rRNA gene sequencing. Consistent with the findings in sheep [14], our results showed significant decreases in the concentrations of total VFA and the proportions of acetate in the rumen fluid of grazing kids compared with kids under indoor feeding, likely due to lower digestibility and less total energy of the diet for the grazing goats. However, insignificant differences in the ratios of acetate/propionate indicated that the rumen fermentation pattern did not change substantially among the three feeding systems, which could be attributed to the high-forage dietary used in our study.

We observed a significantly higher alpha diversity of the rumen bacterial and archaeal populations in the grazing goats and the grazing goats supplemented with concentrate compared to the goats under indoor feeding. This finding was true for sheep, as evidenced by a comparative analysis showing an increase in rumen bacterial diversity when animals were shifted from non-grazing to grazing diets [14]. Similar changes in rumen bacterial diversity were also found in cattle [31] and sheep [32] during the transition from high-forage to high-grain diets. The PCoA and ANOSIM analyses revealed that the rumen bacterial and archaeal community compositions were more similar between the GR- and SF-fed goats, implying that a long grazing period determined the rumen bacterial community. In summary, these findings suggest that adapting to a feeding system with a high proportion of forage requires more bacterial populations, which can be attributed to the need to utilize the complex forage types.

Similar to the findings for the rumen of other goat breeds [8,9,20,33] and cattle [34], the predominant bacterial phyla identified in our study included Bacteroidetes, Firmicutes, Tenericutes and Proteobacteria. As two major phyla in the rumen microbiota, large fluctuations in the Firmicutes/Bacteroidetes ratio have been associated with changes in the relative amounts of dietary forages in goats [18,20] and cattle [31,35]. However, we did not observe a significant difference in this ratio, as reported in sheep [14]. This result might reflect that the starch in the indoor feeding diet was partially compensated by higher soluble carbohydrate content of fresh forage during grazing. Euryarchaeota is generally the predominant phylum in the ruminal archaeal community [4,9,36], as confirmed by our results. Notably, the phylum Gracilibacteria in the domain Bacteria was also identified, and was highly abundant via specific 16S rRNA primers for methanogens (data not shown). This finding was supported by high levels of Gracilibacteria in the methane-enriched environmental samples [37,38], and similarities between the genome sequences of Gracilibacteria and those of archaea [39,40]. Our results showed that *Methanobrevibacter* was the most abundant genus in the rumen archaeal community of ruminants [5], such as goats [20], cattle [41,42], yaks [15] and impala [43].

In the present study, we identified specific microbial taxa (i.e., biomarkers) associated with different feeding systems using LEfSe [28]. Strikingly, the phylum Synergistetes was highly abundant in the rumen, and was identified as a biomarker in grazing goat kids. The members of Synergistetes were first isolated from the goat rumen [44], and this phylum mainly includes bacteria that degrade amino acids [45]. Ito et al. demonstrated that a bacterium belonging to Synergistetes has a higher utilization rate of acetate compared with *Methanosaeta* [46]. However, Synergistetes has not been reported in the rumen of cattle or goats to date, except for one study on Shaanbei white-cashmere goats [8]. These results suggest that colonization of Synergistetes may be host-specific, and its occurrence in the rumen is likely related to local environments. The bacterial biomarkers of the grazing goats included here mainly contained the phylum Planctomycetes and its members. Although this phylum is generally considered to be environmental microorganisms, a few studies showed the existence of Synergistetes in the gut of humans [47,48,49] and termites [50,51].

Cellulolytic bacteria in the rumen have been investigated extensively [52,53] considering their important functions in the digestion of fibrous feedstuffs that cannot be degraded by the host. Previous studies have provided evidence for cellulose-degrading bacteria as biomarkers (e.g., *Lachnospiraceae*) in grazing goats. For instance, because it is enriched in *endo-1*, 4-betaxylanase and cellulase genes [54], *Lachnospiraceae*, belonging to Firmicutes, is highly specialized in the degradation of complex plant material [54,55,56]. Compared to feeding a total mixed ration, the relative abundance of *Veillonellaceae* was about three times higher in pasture-fed cows [13]. We also observed that *Butyrivibrio fibrisolven* and *rumen bacterium NK4A214* were significantly higher in abundance in grazing kids than that in goat kids under indoor feeding, which was in agreement with observations in grazing sheep [57] and yaks [15]. Moreover, isobutyrate or isovalerate supplementation resulted in higher levels of *Butyrivibrio fibrisolven* in the rumen of dairy calves [58] and steers [59], as supported by a previous finding [60].

Here, several archaeal taxa were also detected as biomarkers of the goats under the different feeding systems. For example, the relative abundances of the methylotrophic genus *Methanosphaera* [61] and the species *Methanosphaera sp. ISO3 F5* in the grazing and concentrate-supplemented goat kids were significantly higher than those in the goats under indoor feeding, and similar results were found in Liuyang black goat kids [30]. As a major methanogen in ruminants on different diets [62], the hydrogenotrophic methanogen *Methanobrevibacter* [63] was deemed as a biomarker for goat kids under indoor feeding in our study. However, *Methanobrevibacter ruminantium*, belonging to *Methanobrevibacter*, was significantly associated with the grazing kids. Considering its importance in ruminants, Ufnar et al. proposed *Methanobrevibacter ruminantium* as an indicator of domesticated-ruminant fecal pollution in environmental samples (e.g., surface waters) [64]. Microarray analyses also suggested that an upregulation of methanogenesis genes occurred in this species during co-culture with a hydrogen-producing rumen bacterium [65], thereby providing new insight into the biology of methanogens.

In summary, our study demonstrated that hydrogenotrophic (e.g., *Methanobrevibacter*) [63] and methylotrophic methanogens (e.g., *Methanosphaera*) [61] were mainly enriched in different feeding systems, respectively, indicating that the metabolic pathways for methane changes across feeding systems.

Considering that short-chain fatty acids (i.e., VFAs) are the main end products from the carbohydrate catabolism of gut microbes, we sought to explore correlations between the microbial biomarkers identified above and VFAs in the rumen fluid. Similar to the previous findings in goats [18,66] and sheep [14], most microbes were not significantly correlated with VFAs in this study, mainly due to the complex relationships between diet composition, the gut microbiota and metabolic outputs [55,67] (e.g., substrate cross-feeding [68]). However, linear correlations were observed between several microbial taxa (e.g., *Veillonellaceae* and *Succiniclasticum*) and VFAs, which were supported by previous work. For example, an unclassified genus of *Veillonellaceae* was negatively correlated with propionate concentrations in lambs fed a linseed oil-supplemented diet [69]. Sandri et al. reported that *Veillonellaceae* was negatively correlated with butyrate in lactating cows [70]. Furthermore, the acetate concentration was negatively related to *Succiniclasticum* in dairy cows fed high-grain diets [71], which was consistent with our findings in goats under indoor feeding.

It is noted that the gut microbiota can change with the season in wild animals and grazing ruminants due to seasonal fluctuations of vegetation resources and grass yield in grassland, supported by findings in fecal samples of wild baboons [72], great apes [73] and humans in a hunter-gatherer setting [74]. Based on fecal samples of yaks and Tibetan sheep in the same grazing systems, Wei et al. demonstrated that seasonal diets had a higher impact on the gut microbiota than that of host species [75]. Thus, the effects of seasonal diets on the rumen microbiota of grazing goats deserve to be further investigated.

## 5. Conclusions

In this study, we characterized the rumen microbiota and volatile fatty acid profiles of weaned goat kids under shrub-grassland grazing and indoor feeding. Our results demonstrated that the adaptation to grazing and supplementary feeding required more rumen bacterial populations due to complex dietary sources, although the rumen fermentation pattern did not change substantially among the three feeding systems. Some microbial taxa were considered to be biomarkers associated with one feeding system, particularly cellulose-degrading bacteria. Taken together, our study provides insight into the microbial community and VFA profiles across different feeding systems.

## Figures and Tables

**Table 1:** Concentrations of volatile fatty acids (VFAs) in the rumen fluid of the goat kids among the three feeding systems.

VFAs	GR	SF	IF	*p*-Value
Total VFA (mM)	17.09 ^b^	24.72 ^b^	46.68 ^a^	<0.01
Acetate (molar%)	67.13 ^b^	69.49 ^ab^	72.77 ^a^	0.011
Propionate (molar%)	17.28	15.57	17.09	0.505
Butyrate (molar%)	9.21 ^ab^	10.33 ^a^	7.66 ^b^	<0.01
Iso-butyrate (molar%)	2.43 ^a^	1.69 ^b^	0.86 ^c^	<0.01
Valerate (molar%)	0.95 ^a^	0.64 ^b^	0.54 ^b^	0.015
Iso-valerate (molar%)	2.99 ^a^	2.28 ^a^	1.07 ^b^	<0.01
Acetate: propionate	4.00	4.55	4.36	0.492

Note: Values with different letter superscripts within a row mean significant difference (*p* < 0.05). The same as below.

**Table 2:** Alpha diversities of bacteria and archaea in the rumen fluid of the goat kids among the three groups.

Item	Bacteria				Archaea			
	GR	SF	IF	*p*-Value	GR	SF	IF	*p*-Value
Observed species	93 ^a^	93 ^a^	59 ^b^	0.02	142.67	151.50	119.00	0.06
Shannon	5.49 ^ab^	5.88 ^a^	4.56 ^b^	0.04	3.71	3.49	3.35	0.21
Simpson	0.93	0.97	0.90	0.21	0.85	0.80	0.81	0.40
Chao1	215.04	148.79	99.21	0.18	169.05 ^a^	177.80 ^a^	131.56 ^b^	0.01

## Supplementary Materials

The following are available online at https://www.mdpi.com/2076-2615/10/2/176/s1, Figure S1: The taxonomic cladograms of the bacterial and archaeal biomarkers identified in pairwise comparisons of the three feeding systems, Figure S2: Spearman correlations between archaeal biomarkers and VFAs, Table S1: Ingredient and chemical composition of the concentrate supplement in the IF and SF groups, Table S2: Summary of 16S rRNA gene sequencing reads across samples, Table S3: The ANOSIM analysis of similarity of bacterial and archaeal populations among the three groups, Table S4: The relative abundances of bacterial and archaeal taxa in the rumen of the goat kids among the three groups.
